# Effectiveness of Direct Oral Anticoagulants and Vitamin K Antagonists in Preventing Stroke in Patients With Atrial Fibrillation and Liver Cirrhosis: A Systematic Review and Meta-Analysis

**DOI:** 10.7759/cureus.62606

**Published:** 2024-06-18

**Authors:** Tanya Sinha, Mandeep Kaur, Abshiro H Mayow, Thin M Soe, Khaldoun Khreis, Sandipkumar S Chaudhari, Samer Kholoki, Shamsha Hirani

**Affiliations:** 1 Internal Medicine, Tribhuvan University, Kathmandu, NPL; 2 Hospital Medicine, HCA Capital Regional Medical Center, Tallahassee, USA; 3 Medicine, St. George's University School of Medicine, Chicago, USA; 4 Medicine, University of Medicine 1, Yangon, Yangon, MMR; 5 Pediatrics, Pécs Medical University, Pécs, HUN; 6 Cardiothoracic Surgery, University of Alabama at Birmingham, Birmingham, USA; 7 Family Medicine, University of North Dakota School of Medicine and Health Sciences, Fargo, USA; 8 Internal Medicine, La Grange Memorial Hospital, Chicago, USA; 9 Cardiology, Baqai Hospital, Karachi, PAK

**Keywords:** liver cirrhosis, atrial fibrillation, stroke, oral anti-vitamin k, direct oral anticoagulant therapy

## Abstract

Patients with atrial fibrillation and concurrent liver cirrhosis have been excluded from major clinical trials evaluating direct oral anticoagulants (DOACs) due to safety concerns. This has led to uncertainty regarding the optimal anticoagulant therapy in this population at high risk of thromboembolic events. We conducted a systematic review and meta-analysis to compare the effectiveness and safety of DOACs versus vitamin K antagonists (VKAs) in patients with atrial fibrillation and liver cirrhosis. Databases including Embase, MEDLINE/PubMed, and Web of Science were searched for relevant studies. The primary effectiveness outcome was stroke or systemic embolism, and the safety outcome was major bleeding events. A total of 10 studies were included in the meta-analysis. Compared to VKAs, the use of DOACs was associated with a significantly lower risk of stroke or systemic embolism (RR: 0.78, 95% CI: 0.65-0.92, p=0.005). The risk of all-cause mortality was comparable between the two groups (RR: 0.89, 95% CI: 0.74-1.07, p=0.23). Notably, DOACs demonstrated a significantly lower risk of major bleeding events (RR: 0.67, 95% CI: 0.61-0.73, p<0.01) compared to VKAs. This meta-analysis suggests that DOACs may be a favorable alternative to VKAs for the prevention of thromboembolic events in patients with atrial fibrillation and liver cirrhosis, with a lower risk of stroke or systemic embolism and major bleeding. However, further research is needed to establish optimal dosing strategies and assess the safety and efficacy of DOACs in patients with advanced liver disease.

## Introduction and background

In the last 10 years, the incidence of cirrhosis in the United States has doubled, and the rates of cirrhosis-related hospitalizations and deaths are expected to triple by 2030 [1, 2]. Atrial fibrillation and its thromboembolic complications affect up to 15% of cirrhosis patients, resulting in significant additional morbidity and mortality [3]. Globally, the burden of atrial fibrillation is rising due to increased life expectancy [4]. Patients with atrial fibrillation face a fivefold increased risk of stroke compared to the general population. Anticoagulant therapy is essential in managing atrial fibrillation patients to prevent thromboembolic events, especially strokes [5].

Warfarin has been the most widely used oral anticoagulant for preventing ischemic strokes and venous thromboembolic events. Although vitamin K antagonists (VKAs) like warfarin are effective anticoagulants, they have several drawbacks [6]. These include variability in individual doses, a narrow therapeutic window, the need for regular monitoring, and sensitivity to both dietary and pharmacological factors that can affect their efficacy [7]. Direct oral anticoagulants (DOACs) offer an alternative option for individuals with atrial fibrillation and have increasingly supplanted VKAs in clinical practice. For the majority of adults with atrial fibrillation, DOACs provide a significant net clinical benefit in preventing major thromboembolic events [8, 9]. They are favored for their broader therapeutic window and ease of use, owing to fixed oral dosing and reduced need for regular laboratory monitoring or dose adjustments compared to VKAs [10].

However, patients with atrial fibrillation and concurrent liver cirrhosis have consistently been excluded from key randomized controlled trials due to safety concerns. The ideal oral anticoagulant therapy (OAT) for cirrhotic patients at high risk of thromboembolism remains a subject of debate. Despite this, DOACs have gained traction and are now recommended in the BAVENO guideline for patients with Child-Turcotte-Pugh (CTP) A and B cirrhosis [11]. Different studies have compared DOACs in patients with liver cirrhosis [12-21]. However, much of the evidence supporting the use of DOACs in atrial fibrillation patients with liver cirrhosis has primarily come from studies with small sample sizes [12, 13, 15, 17, 18]. A previous systematic review endorsed the use of DOACs in patients with atrial fibrillation and liver cirrhosis, but several new studies have emerged since [12-14, 20]. Thus, this study was needed to compare the effectiveness and safety of DOACs to prevent stroke with VKAs in these patients.

## Review

Methodology 

The "Preferred Reporting Items for Systematic Reviews and Meta-Analyses" (PRISMA) criteria were followed in the conduct of this systematic review and meta-analysis.

We searched the databases Embase, MEDLINE/PubMed, and Web of Science for publications published between the databases' launch date and May 15, 2024. The terms "atrial fibrillation," "liver cirrhosis," "vitamin K antagonists," and "oral anticoagulants," along with their synonyms and medical subject heading (MeSH) terms, were utilized to search for pertinent papers. To find more publications, a bibliographic search of relevant reviews and included papers was carried out. Two authors conducted the search independently. When disagreements arose during the search, they were discussed, and if necessary, the primary investigator got involved.

Study Selection 

Two independent reviewers conducted the study screenings, with a third reviewer resolving any discrepancies through consensus. Initially, titles and abstracts were screened, followed by full-text publications. Those meeting the inclusion criteria were selected for data extraction. 

Our inclusion criteria encompassed all clinical study designs, including prospective, retrospective, and randomized clinical trials, that compared at least one of the outcomes evaluated in this investigation. There were no language restrictions on the articles. The DOACs included were edoxaban, apixaban, rivaroxaban, and dabigatran. The effectiveness outcomes assessed in this meta-analysis were ischemic stroke, transient ischemic attack (TIA) or systemic embolism, and all-cause mortality. Major bleeding was the safety outcome. We excluded case reports, reviews, and other publications lacking sufficient data. 

Data Extraction 

Two authors performed the process of data extraction independently. A standardized data extraction form created in Microsoft Excel was used to collect pertinent data directly from each publication. The following information was extracted: authors, publication year, geographic location, study design, sample size, study groups, length of follow-up, and study outcomes. Disagreements between the two authors were resolved through discussion.

Statistical Analysis 

RevMan 5.4.1 was employed to perform quantitative pooling and analysis. I-square values were used to measure the heterogeneity among the studies; an I-square of less than 50% indicated low heterogeneity, while an I-square of more than 50% indicated high heterogeneity. A fixed-effects model was used for analyses with low heterogeneity, and a random-effects model was used for those with significant heterogeneity. The risk ratios (RR) and 95% confidence intervals (CI) for the meta-analysis results are presented. A p-value of less than 0.05 was considered statistically significant.

Results

**Figure 1:**
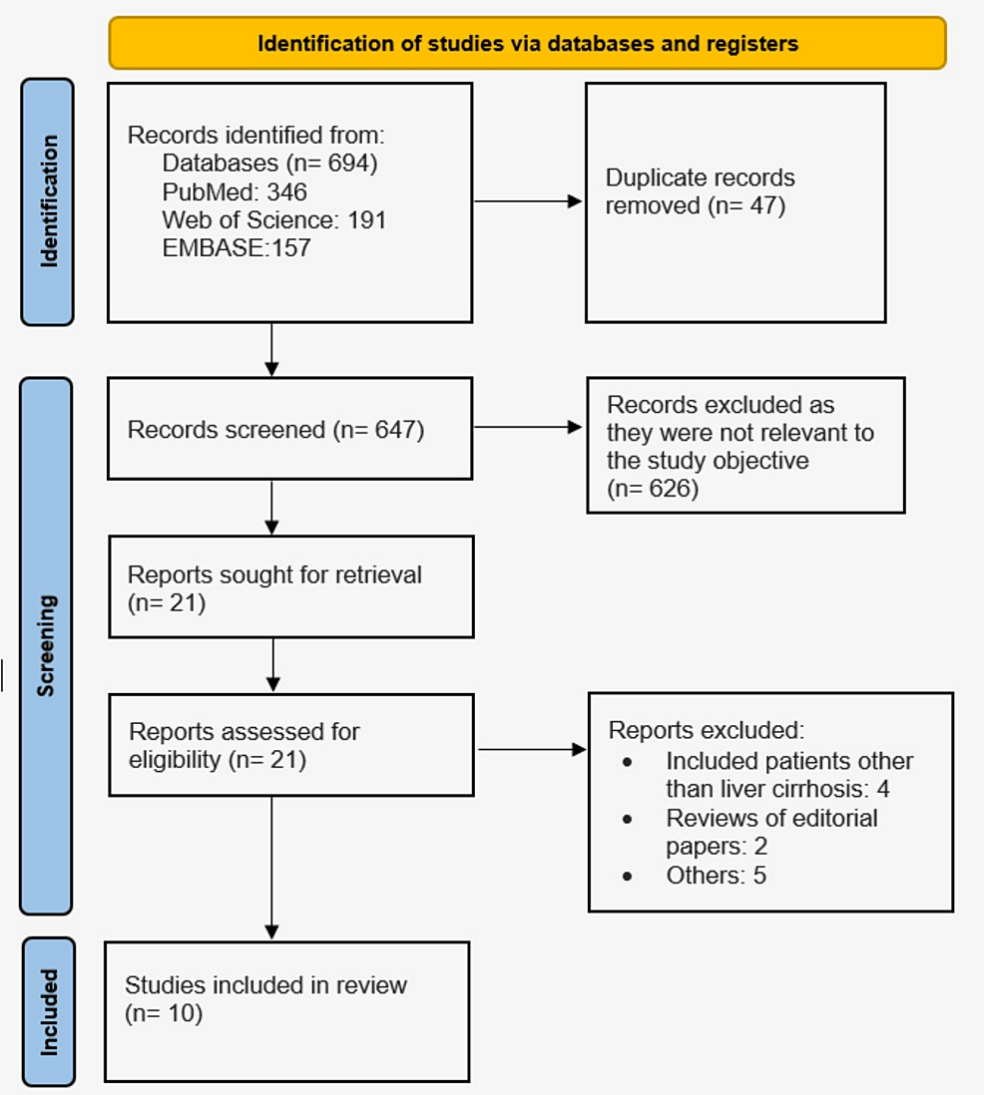
PRISMA flowchart of included studies

A total of 694 papers were found during the literature search. After duplicates were eliminated, 647 articles' titles and abstracts were examined. Of these, 626 pieces were discarded, and the eligibility of the remaining 21 articles was evaluated in their entirety. The meta-analysis includes data from the 10 publications that remained after an additional 11 articles were eliminated. The PRISMA 2020 flow diagram's research selection flow chart is depicted in Figure 1. Table 1 presents the characteristics of the included studies.

**Table 1:** Characteristics of included studies DOAC: direct oral anticoagulant; VKA: vitamin K antagonist; NS: not specified.

Author ID	Year	Study Design	Region	Groups	Sample Size	Length of Follow-up
Baylo et al. [12]	2023	Retrospective	Ukraine	DOAC	30	NS
				VKA	26	
Chou et al. [13]	2023	Retrospective	Taiwan	DOAC	478	3.2 years
				VKA	247	
Douros et al. [14]	2024	Retrospective	Canada and England	DOAC	1811	NS
				VKA	872	
Goriacko et al. [15]	2018	Retrospective	United States	DOAC	75	NS
				VKA	158	
Lawal et al. [16]	2023	Retrospective	United States	DOAC	1399	0.57 years
				VKA	1541	
Lee et al. [17]	2019	Retrospective	Taiwan	DOAC	1397	1.3 years
				VKA	946	
Lee et al. [18]	2019	Retrospective	Taiwan	DOAC	446	1.2 years
				VKA	322	
Serper et al. [19]	2021	Retrospective	United States	DOAC	201	4.6 years
				VKA	614	
Song et al. [20]	2023	Retrospective	United States	DOAC	384	NS
				VKA	275	
Yoo et al. [21]	2022	Retrospective	Korea	DOAC	128	7.3 years
				VKA	110	

Primary Efficacy Outcome (Stroke or Systemic Embolism) 

**Figure 2:**
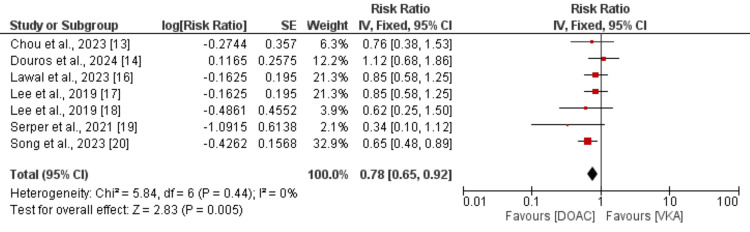
Comparison of stroke or standard error (SE) between DOAC and VKA DOAC: direct oral anticoagulant; VKA: vitamin K antagonist. References [13-14, 16-20].

As shown in Figure 2, seven studies compared the risk of developing stroke or systemic embolism between DOAC and VKA. Compared to VKA, the risk of developing stroke or systemic embolism was significantly lower in patients receiving DOAC (RR: 0.78, 95% CI: 0.65 to 0.92, p = 0.005). No significant heterogeneity was found among the study results (I-square: 0%).

Secondary Efficacy Outcome (All-Cause Mortality) 

**Figure 3:**
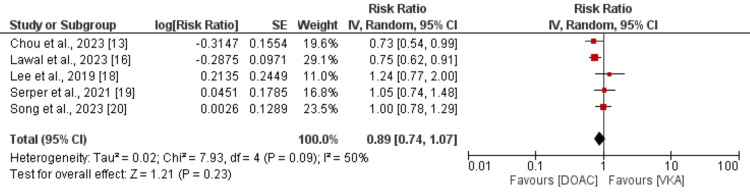
Comparison of all-cause mortality between DOAC and VKA DOAC: Direct oral anticoagulant; VKA: Vitamin K antagonist; SE: standard error. Sources: References [13, 16, 18-20].

The pooled analysis, which compared the risk of all-cause death between the two groups, included five studies. The findings are shown in Figure 3. Compared to VKA, patients taking DOAC had an 11% decreased risk of all-cause mortality, although the difference was not statistically significant (RR: 0.89, 95% CI: 0.74 to 1.07, p = 0.23). The studies showed significant heterogeneity (I-square: 50%). 

Safety Outcome (Major Bleeding Events) 

**Figure 4:**
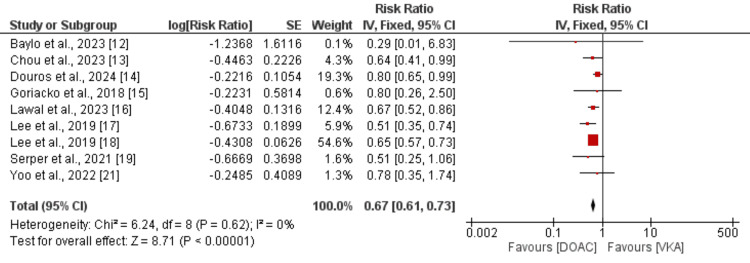
Comparison of major bleeding events between DOAC and VKA DOAC: direct oral anticoagulant; VKA: vitamin K antagonist; SE: standard error. References [12-19, 21].

Figure 4 presents the pooled analysis results for the nine studies that reported significant bleeding incidents. According to the pooled analysis, patients in the DOAC group had a significantly lower risk of major bleeding events (RR: 0.67, 95% CI: 0.61 to 0.73, p < 0.01) than those in the VKA group. There was no evidence of significant heterogeneity (I-square: 0%).

Discussion 

In the present meta-analysis, we found that DOAC treatment is associated with a lower risk of stroke or systemic embolism compared to VKA in patients with atrial fibrillation and liver cirrhosis. The rate of mortality was comparable between the two groups. Additionally, the risk of major bleeding events also appeared to be lower in the DOAC group. A previous meta-analysis conducted by Hu et al. [6] reported a lower risk of stroke or systemic embolism and all-cause mortality in patients with atrial fibrillation and liver cirrhosis, although the difference was statistically insignificant. However, DOACs are associated with more favorable safety outcomes. These findings suggest that DOACs can be a feasible choice of anticoagulant in patients with liver cirrhosis and atrial fibrillation.

Liver cirrhosis patients exhibit reduced synthesis of coagulation factors as well as anticoagulation proteins [22]. Consequently, cirrhosis increases the risk of both bleeding and clotting complications [23]. Although conditions like portal hypertension and hypersplenism can cause thrombocytopenia in advanced cirrhosis patients, bleeding due to thrombocytopenia is rare. This is because of a simultaneous decrease in von Willebrand factor (vWF)-cleaving ADAMTS13, which leads to elevated vWF levels that promote platelet activation [24]. Unfortunately, commonly used hemostasis markers such as prothrombin time and platelet count are inadequate for predicting bleeding risk in cirrhosis patients, as they do not reflect the reduction in coagulation proteins [25]. The uncertainty regarding bleeding risk has resulted in the exclusion of cirrhosis patients from key clinical trials comparing DOACs and warfarin.

Therefore, this meta-analysis aims to evaluate the suitability of DOACs for use in patients with cirrhosis. As this meta-analysis shows improved outcomes with DOACs, they can be considered an alternative option to warfarin due to the lower risk of adverse events in patients with liver cirrhosis and atrial fibrillation.

Furthermore, research has demonstrated that compared to warfarin users, patients with atrial fibrillation taking DOACs experienced better health-related quality-of-life scores [26]. This could be due to warfarin's limited therapeutic range, which necessitates regular dose modifications and coagulation monitoring [27]. Additionally, maintaining the therapeutic range of warfarin becomes increasingly difficult due to the growing list of medications and foods that negatively interact with it [28]. The management of various comorbidities may be significantly impacted by these other factors to consider when using warfarin. Furthermore, due to drug-food interactions, patients using warfarin must restrict their diet, which may lower their quality of life. Because they allow for a higher quality of life, some atrial fibrillation patients choose DOACs over warfarin [29].

In atrial fibrillation patients with liver cirrhosis, the use of DOACs instead of VKAs will benefit in terms of the prevention of stroke and bleeding events. These findings refer to patients with preserved liver function. Despite being contraindicated in CP B cirrhotic individuals, edoxaban and rivaroxaban have been used in certain studies with no notable side effects, according to the Food and Drug Administration. Due to a lack of data, there is currently no conclusive evidence about the optimal DOAC agent. Dabigatran, however, seems like a sensible option due to its renal clearance [30]. However, treatment decisions should be tailored to each patient, considering potential drug interactions, kidney function, and liver health. It is important to note that much of the existing data comes from retrospective studies and includes only a limited number of patients with advanced cirrhosis. Current evidence indicates that lower doses of DOACs might be both safe and effective for patients with liver disease. Nonetheless, the optimal dose adjustment for DOACs in cirrhotic patients with atrial fibrillation still needs to be established.

Study Limitations 

The current meta-analysis has several limitations. Firstly, all the included studies were observational. Notably, all randomized controlled trials (RCTs) comparing DOACs with VKAs excluded patients with liver cirrhosis. Future trials are needed to evaluate the effects of DOACs in this specific population. Secondly, the Child-Pugh classification was available in only three publications, with most patients falling into class A or B, and therefore we were unable to perform a subgroup analysis. The use of DOACs in patients with atrial fibrillation who are classified as Child-Pugh class C also requires further investigation. Thirdly, we were unable to assess the quality of INR control for patients on warfarin. Consequently, the less favorable outcomes for warfarin compared to DOACs may be attributed to poor INR management.

## Conclusions

In conclusion, this meta-analysis demonstrates that DOACs are associated with a lower risk of stroke or systemic embolism and major bleeding events compared to VKAs in patients with atrial fibrillation and liver cirrhosis. The all-cause mortality risk was comparable between the two groups. These findings suggest that DOACs could be a favorable option for anticoagulation in this patient population. However, further research, including randomized controlled trials, is warranted to establish the optimal DOAC agent and dosing strategies, particularly in patients with advanced liver disease.
